# Multi-Scale Structure and Directional Hydrophobicity of Titanium Alloy Surface Using Electrical Discharge

**DOI:** 10.3390/mi13060937

**Published:** 2022-06-12

**Authors:** Mengjie Wang, Zilong Peng, Chi Li, Junyuan Zhang, Jinyin Wu, Fei Wang, Yinan Li, Hongbo Lan

**Affiliations:** 1 Shandong Engineering Research Center for Additive, Qingdao University of Technology, Qingdao 266520, China; wangmengjie9696@163.com (M.W.); lclc1225@163.com (C.L.); zhangjunyuan980414@163.com (J.Z.); wujinyin1215@163.com (J.W.); wangfei@qut.edu.cn (F.W.); liyinan@qut.edu.cn (Y.L.); hblan99@126.com (H.L.); 2 Key Lab of Industrial Fluid Energy Conservation and Pollution Control (Qingdao University of Technology), Ministry of Education, Qingdao 266520, China; 3 Shaanxi Special Equipment Inspection and Testing Institute, Xi’an 710000, China

**Keywords:** titanium alloy, surface, WEDM, HV-μEAM, macro-micro composite structure, directional hydrophobicity

## Abstract

Titanium alloys with special macro-micro composite structures of directional hydrophobicity are difficult to prepare due to poor thermal conductivity and good corrosion resistance, inhibiting the wide engineering applications for aerospace, marine engineering, and biomedicine. To prepare macro-micro composite structures on the surface of titanium alloys and achieve directional hydrophobicity, the sub-millimeter structures with an edge width of 150 μm, a groove width of 250 μm, and a depth of 250 μm were fabricated on the titanium alloy by wire electrical discharge machining (WEDM) technology, and high voltage-induced weak electric arc machining (HV-μEAM) was used to fabricate micro-scale feature size micro-structures on the processed macro-structure edges. The influence of process parameters on the morphology of microstructures was studied experimentally. The smooth surface of the titanium alloy is isotropically hydrophilic, and its contact angle is 68°. After processing the macrostructure on the titanium alloy surface, it shows directional hydrophobicity after being modified by low surface energy materials. The macro-micro composite structure formed by HV-μEAM realizes a directional hydrophobic surface with contact angles (CA) of 140° (parallel direction) and 130° (perpendicular direction), respectively. This surface has been modified with low surface energy to achieve contact angles of 154° and 143°. The results of the abrasion resistance test show that under the load of 100 g, it retains directional hydrophobicity at a friction distance of 700 mm with 600# sandpaper. The existence of the sub-millimeter macrostructure is the reason for the directionality of surface hydrophobicity. The microstructure can realize the transformation of the titanium alloy surface from hydrophilic to hydrophobic. Under the combined effects of the macro and micro composite structure, the surface of the titanium alloy shows obvious directional hydrophobicity.

## 1. Introduction

The study of the surface wettability properties [1] of materials has largely been derived from observing the unique structures of the body surfaces of plants and animals in nature, such as the self-cleaning properties of lotus leaves [2,3,4,5] and the high adhesion of rose petals [6,7,8,9]. These properties are attributed to their unique Macro-micro composite structures and their combined forms. Materials exhibit different hydrophobic properties in different directions with significant differences in the anisotropic CA. This property is known as anisotropic hydrophobic surfaces [10,11,12,13]. This property is common in biological surface structures. For example, butterfly wings in biology [14,15,16] are typical examples of directional hydrophobic surfaces achieved through macro-micro composite structures. The micron-sized square scales on the surface of the butterfly’s wings have a nanoscale riblet structure, so water droplets on the surface roll off in the outward direction of the body. The water droplets cannot roll off in the opposite direction because of the high adhesion force and thus, roll off the next time the butterfly swings its wings. This exhibits anisotropic hydrophobicity. The surface of rice leaves [17,18,19] is also an anisotropic hydrophobic surface. It has a sub-millimeter groove array structure of large size. A large number of micrometer-scale papilla structures are also distributed on the groove ribs. The papillae structures are filled with nanoscale needle-like forms. The presence of this macro-micro composite structure on rice leaves makes it easier for water droplets on rice leaves to roll in a direction parallel to the edge of the leaf.

So far, various methods have been tried to prepare macro-micro composite structures to achieve anisotropic hydrophobic surfaces, involving micromachining and micro-special machining specialties. The processed materials cover non-metallic materials such as glass and metallic materials such as aluminum alloy and stainless steel. Lian et al. [20] processed a groove macrostructure with 100 μm spacing on the aluminum alloy surface by nanosecond laser, combined with the microstructure generated by laser spot during laser treatment. They successfully prepared a superhydrophobic aluminum alloy surface with an anisotropic surface. Zhang et al. [21] processed grating microarray structures with intervals of 200 μm on the surface of an aluminum alloy substrate by a high-speed micro-milling method, and there were many irregular nanoscale pits and bumps on the surface of the submillimeter grating. Under the joint action of such macro-micro composite structures, the aluminum alloy surface showed anisotropic hydrophobic properties. Yunusa et al. [22] bonded tubular polymer fibers with a diameter of 300 μm on the surface of the glass and other workpiece materials in a certain order and obtained anisotropic superhydrophobic surfaces after spraying with nano-silica. Patel et al. [23], prepared hydrophobic surfaces by using a NaNO_3_ solution as electrolyte and electrochemical micromachining (ECMM) and mask technology to fabricate microgroove structures with a depth of 70~200 μm and a width of 250~300 μm on the stainless steel surface. Wang et al. [24] used a combination of topography structure and chemical coating to construct a superhydrophobic surface on titanium alloy implants and successfully constructed a layered surface with microroughness and nanotubes. It has a contact angle (CA) of 44.9°. After being modified by fluoroalkylsilane, the contact angle was 151.4°, which exhibited superhydrophobicity.

Titanium alloy [25] is often used in the manufacture of micro and precision parts due to its advantages of low density, high specific strength, and good corrosion resistance. It is widely used in aerospace [26,27], ocean engineering [28], biomedical [29,30], and other fields. The anti-oxidation, wear reduction, and corrosion resistance of titanium alloys with superhydrophobicity has been substantially improved. In aerospace, superhydrophobic titanium alloys improve mechanical durability. In marine engineering, superhydrophobic titanium alloys prevent corrosive media from coming into contact with the metal, reducing galvanic corrosion and increasing service life. In biomedicine, the superhydrophobic surface of implanted titanium alloys facilitates the reduction of alloy-blood cell interactions, preventing bacterial adhesion and reducing the risk of infection. However, the study of its surface wettability characteristics is more important. Directional hydrophobicity [31,32,33] can move droplets in a directional manner by setting special macro-micro composite structures and their combined forms. It has special applications in directional drag reduction. 

Because of the poor thermal conductivity of titanium alloy, the tool wear will be intensified during mechanical processing, and it is difficult to remove the material during special machining [34]. Spinwall et al. [35] studied the titanium alloy surface processed by wire electrical discharge machining (WEDM), where good surface quality was obtained using high pulses and low pulse widths. These phenomena are more prominent in micromachining. Therefore, research on the preparation of anisotropic hydrophobic surfaces in a titanium alloy matrix is relatively rare. Peng et al. [36] Proposed a high-voltage weak arc processing (HV-μEAM) additive manufacturing method, which can deposit complex trajectories and form micron-level metal precipitates on the workpiece. The deposition is dense and continuous. In this paper, we proposed an electrical discharge machining-based surface processing method for macro-micro composite structures. Wire WEDM technology was used to machine sub-millimeter groove macrostructures. The high voltage induced weak electric arc machining HV-μEAM was used to fabricate micro-scale feature size micro-structures on the processed macro-structure edges. After further low surface energy treatment, the hydrophobicity of the titanium alloy was further improved, and the superhydrophobic effect was achieved.

## 2. Materials and Methods

### 2.1. Materials and Characterization Methods

The substrate material for the TC4 titanium alloy, the chemical composition of which is shown in Table 1, was purchased from Dongguan Guangyue Metal Materials Co., Ltd. (Dongguan, Guangdong, China). The chemical reagents in the experiment—acetone and anhydrous ethanol—are analytically pure, and the deionized water and perfluorodecyltriethoxysilane (hereinafter referred to as fluorosilane) were purchased from Guangzhou Hongcheng Biotechnology Co. Ltd., (Guangzhou, Guangdong, China).

A scanning electron microscope model MERLIN Compact from Carl Zeiss, Germany, was used to observe the microstructure of the titanium alloy surface. The static contact angle was measured using a contact angle measurement model JC2000C1 of Shanghai Zhongchen Digital Technology Equipment Co., Ltd. (Shanghai, China). For each measurement, 4 μL of ionized water drops were placed on the surface of the specimen at four different locations, and the average value was taken as the measurement result.

### 2.2. Macro-Micro Composite Structure Processing Method

**Figure 1:**
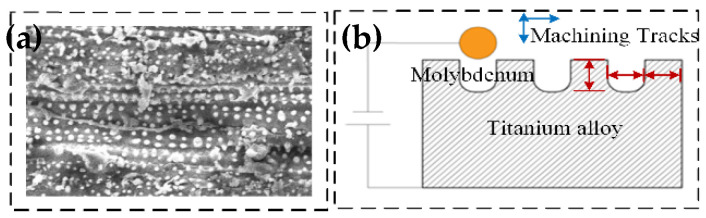
Macrostructure processing schematic diagram. (**a**) Rice leaf and SEM image of macro-micro complex structure on the leaf surface. Reproduced with permission from Ref. [37] (**b**) Machining tracks of macrostructure.

Macro-micro composite structures are an effective means to achieve directional hydrophobic surfaces. By analogy with the microstructure of rice leaves, the surface has a multi-level composite structure consisting of sub-millimeter grooves and micro-nano-scale papilla, as shown in Figure 1a. The macrostructure is a groove structure with a width of several hundred micrometers and a height of several tens of micrometers. For the fabrication of the macrostructure, the sub-millimeter groove structure was processed by WEDM technology in this paper. Figure 1b shows the schematic diagram of the wire-cutting process, the design dimensions of the slot width were 250 μm, edge width b of 150 μm, and slot depth h of 250 μm. The cut surface was polished to reduce the effect of discharge surface etching pits on the analyzed structure. The specimens were ultrasonically cleaned in acetone, anhydrous ethanol, and deionized water, then dried and set aside.

The WEDM process uses the heat generated instantaneously by the high-frequency pulse discharge between the negative electrode (molybdenum wire) and the positive electrode (titanium alloy) to remove the material. Therefore, machining parameters such as peak current, pulse width, and pulse interval affect the machining stability, surface topography, and the size of the discharge gap. The relationship between slot width and discharge gap is shown in Formula (1),

a = d + 2δ,(1)

where a is the groove width (μm), d is the diameter of the wire electrode (μm), and δ is the discharge gap (μm).

In order to better process it, we used the orthogonal experimental design [38,39] to experiment with the processing parameters. Wire breakage is prevented by processing conditions with large pulse widths. The pulse widths of the orthogonal test were set to 16, 24, 32, and 40 μs, and the pulse intervals were set to 30, 35, 40, and 45 μs. The current was selected between 3 and 6 A, and the gap voltage was selected between 5 and 8 steps to ensure the integrity of the processing structure (Table 2). 

The orthogonal experimental data were analyzed using range analysis methods, and the results are shown in Table 3. The equation of the range analysis is shown in Formula (2).

(2) $$R_{j\;}=\max\left[\bar{K_{\mathrm{ji}}},\;\bar{K_{\mathrm{ji}}},\;\cdots \right]-\min\left[\bar{K_{\mathrm{ji}}},\;\bar{K_{\mathrm{ji}}},\;\cdots \right]\;(i=1,\;2,\;3,\;4;\;j=1,\;2,\;3,\;4)$$

where K_ji_ is the sum of the test indicators corresponding to the i-th level of the j-th factor; $\bar{K_{\mathrm{ji}}}$ is the average of the sum of the test indicators corresponding to the i-th level of the j-th factor.

The degree of influence on the edge width size in descending order: peak current > pulse width > gap voltage > pulse interval, which indicates that among the electrical parameters, the peak current has the most significant influence on the edge width and the best combination of parameters is A2B1C2D4, with specific parameters: pulse width of 24 μs, pulse interval of 30 μs, peak current of 4 A, and gap voltage of 8 V.

The micro-scale structure is processed by HV-μEAM. The surface micron-level microstructure is constructed by partial discharge on the surface of the prepared titanium alloy groove edge. Micro-nano-scale surface microstructures are processed by using electrical discharge energy between the poles. It is realized by the melting and re-coagulation of the discharge microzone. According to the principle of interelectrode discharge, the discharge gap is proportional to the voltage between poles to achieve low voltage discharge in gas. The gap between the poles needs to be small enough to penetrate the interpolar air medium.

**Figure 2:**
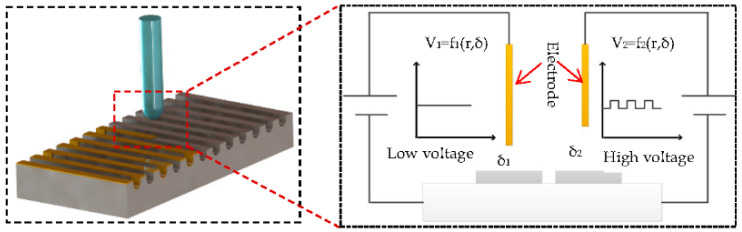
Schematic diagram of high voltage-induced weak arc for microstructure.

As shown in Figure 2, the low voltage discharge gap is δ1. With a high voltage pulse, achieving the breakdown of the discharge medium in the large interpolar gap δ2 can effectively improve the discharge machining gap, forming the plasma channel. At this time, the energy of low voltage and the large current can pass through the plasma channel to achieve local melting of the titanium alloy surface. Along with the machining trajectory of two poles, the melting microzone is recoagulated to form the microstructure. The parameters of HV-μEAM are shown in Table 4.

In order to compare and analyze the effect of each stage of the macro-micro composite structure, the surface was treated with low surface energy solutions. The solution is prepared by mixing fluorosilane with ethanol. For the preparation process of sample parts with low surface energy modification, 0.5 mL of fluorosilane (97%) was dissolved in 50 mL of anhydrous ethanol and stirred well to prepare a low surface energy solution. The sample was immersed in the low surface energy solution for 0.5 h and dried in a 100 °C oven for 2 h. The hydrophobicity of the macro-micro complex structure was comparatively analyzed by analyzing the change of contact angle.

## 3. Results and Discussion

### 3.1. Macrostructure Results

**Figure 3:**
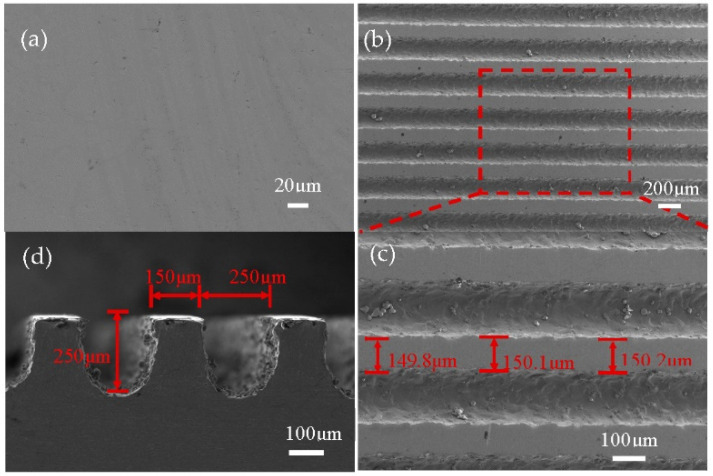
SEM image of groove morphology. (**a**) SEM image of titanium alloy plate. (**b**) Low magnification of the groove. (**c**) High magnification of the groove. (**d**) SEM image of the groove side view.

In this work, the grooves were processed by WEDM, and the macrostructure with better machining accuracy was obtained, as shown in Figure 3. It can be seen that the macrostructure processing was good. The grooves were 250 μm deep, 150 μm wide, and 250 μm wide. Due to the WEDM process, there were discharge etching particles adhered to the surface of the grooves of the macrostructure, impacting the follow-up analysis. Polishing the surface of the groove edge, the smooth groove-edge surface was obtained, which was prepared for the subsequent high pressure-induced weak arc machining of the micro-scale structure.

### 3.2. Microstructure Results

**Figure 4:**
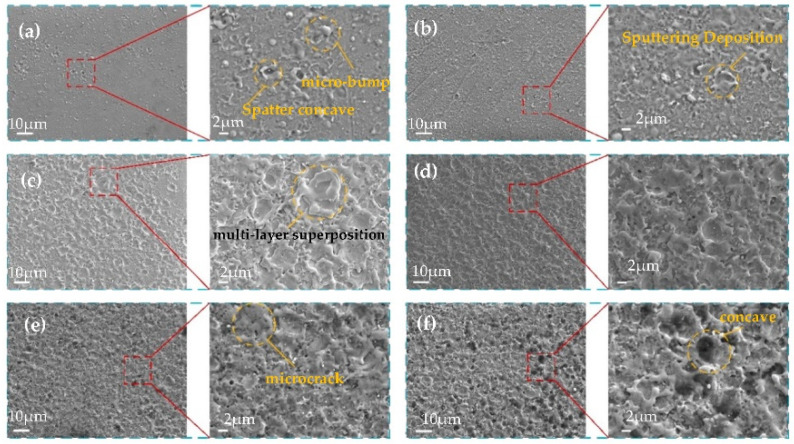
Morphologies of different low-voltage electric current currents: (**a**) 0.5 A; (**b**) 1 A; (**c**) 1.5 A; (**d**) 2 A; (**e**) 2.5 A; (**f**) 3 A.

The morphology and size of the surface microstructure of the material will affect the wettability of the surface. In HV-μEAM machining, machining energy affects the surface morphology; it further affects the wettability of the surface. In this paper, the influence of discharge energy on the micro-scale structure morphology of the machined surface was studied. At the same time, the influence law of surface wettability was obtained. As can be seen from the processing conditions, the effect of interelectrode high pressure increased the ionization degree of the discharge channel, increasing the discharge gap. High voltage and low current machining conditions were adopted. The role of interpolar low pressure is to inject effective energy into the local melting of the titanium alloy surface. The machining conditions of low voltage and high current were adopted. We focused on the study of different input low-voltage electric currents from 0.5 A to 3.0 A. The surface morphology of the titanium alloy groove edge is shown in Figure 4.

Surface roughness seriously affects wettability [40]. The diameter, depth, and density of the surface texture have a significant impact on the contact angle [41]. In HV-μEAM machining, machining energy affects the surface morphology; it further affects the wettability of the surface. 

HV-μEAM is a direct writing process. The local micro material on the surface of titanium alloy melts under the action of discharge energy. When the tool electrode is moved out of the discharge area, the molten microzone rapidly condenses to form a microstructure. This instantaneous non-equilibrium solidification process does not allow sufficient time for the molten material to grow after nucleation, the fine grain microstructure is obtained, and the local sudden cooling and heating lead to increased internal thermal stresses and microscopic cracks. At the same time, the low thermal conductivity of titanium alloy increases the formation of local microcracks.

With a current of 0.5 A, only a small amount of micro-bumps and crater-like structures formed by sputtering can be formed in localized areas, and the titanium alloy substrate can be seen in some areas, as shown in Figure 4a. The density of microtextures is small. As the current increases, irregular bumps appear on the surface of the titanium alloy, and the density of the surface texture increase accordingly, as shown in Figure 4b,c. When the current reaches 2 A, the microtextures start to superimpose, and the surface quality is uniform, forming “islands”. The microtextures not only increase in size and density but also are uniformly distributed on the titanium alloy surface, so the roughness is greatly improved, as shown in Figure 4d. When the low-voltage current is increased to 2.5 A, the energy continues to become larger, the surface quality starts to deteriorate, and micro-cracks appear, as shown in Figure 4e. When the current was increased to 3 A, copper electrodes were ablated, micro texturing cracks increased, and surface quality was poor, as shown in Figure 4e.

From the above microscopic morphology analysis, it can be seen that the HV-μEAM formed microstructures of micron-scale on the smooth titanium alloy surface. With the increase of current, the surface morphology of titanium alloy changes greatly. When the low voltage current was 1.5 A and 2.0 A, the surface had good morphology. The microstructures were in the range of 5 μm and distributed uniformly without large microcracks. The processing requirements of micro-scale structure can be satisfied from scale and micro-morphology.

### 3.3. Titanium Alloy Macro-Micro Composite Surface Directional Hydrophobicity Analysis

#### 3.3.1. Macrostructure Effects on Directional Surface 

**Figure 5:**
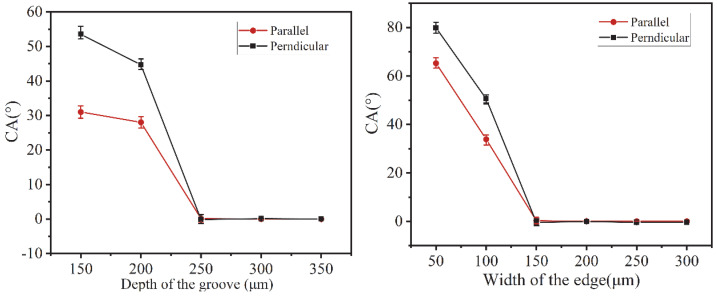
Effect of macrostructure on contact angle. (**a**) Effect of groove depth on contact angle. (**b**) Effect of edge width on contact angle.

To study the effect of macrostructure on surface direction hydrophobicity, the surface of the titanium alloy plate and titanium alloy plate with the macrostructure groove structure was analyzed in comparison by contact angle characterization, and the results are shown in Figure 5. The direction of the groove is called the parallel contact angle, and the direction perpendicular to it is called the perpendicular contact angle.

In order to investigate the relationship between the physical dimension and surface wettability in the macrostructure, highly efficient cutting parameters were used to process the macrostructure. The molybdenum wire diameter plus the discharge gap lead to a slot width of 250 μm (obtained from the test cut machining) while ensuring high machining accuracy. Therefore, the influence of the groove depth and edge width on CA was investigated with a constant slot depth.

The CA measurement results show that when the groove width is 250 μm and the edge width is 150 μm, the CA decreases gradually with the increase of the groove depth, and the contact angle is 0 when the slot depth exceeds 250 μm. The CA measurement results in 2b show that when the groove width is 250 μm and the groove depth is 250 μm, the contact angle decreases with the increase of the edge width, and CA is 0 when the edge width exceeds 150 μm.

The macrostructures with grooves exhibit more anisotropic hydrophilicity, in accordance with the Wenzel infiltration model. The sample with the macroscopic groove structure was modified with the low surface energy material fluorosilane for a more intuitive evaluation. The results exhibit directional hydrophobicity. This indicates that the groove macrostructure has a significant effect on the wettability of the droplets on the titanium alloy surface and that the contact angle of the droplets in the parallel direction is larger than that in the perpendicular direction, further indicating that the presence of the groove macrostructure makes the surface wettability of the titanium alloy directional.

**Figure 6:**
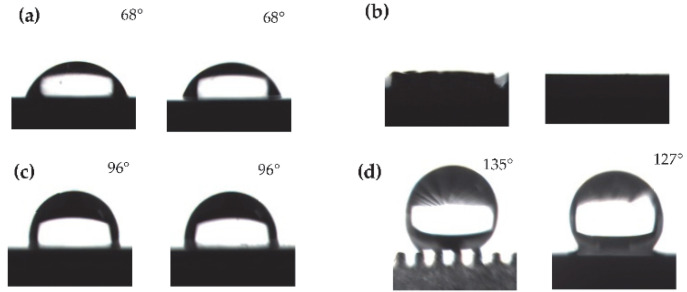
CA of smooth surface and macrostructure surface. (**a**) CA of unprocessed titanium alloy plate; (**b**) surface wettability of macrostructure; (**c**) CA of unmachined titanium alloy plate after low surface energy modification; (**d**) CA of titanium alloy plate with macrostructure after low surface energy modification.

When 4 μL of deionized water droplets were placed on the surface of the unprocessed titanium alloy plate, each CA was 68°, as shown in Figure 6a. At this time, the unprocessed titanium alloy plate exhibited isotropic hydrophilicity in terms of wettability. The results of contact angle measurements on the surface of titanium alloy with macro-structured prismatic grooves are shown in Figure 6b. At this point, the water droplets will spread completely along the grooves and enter the grooves without surface hydrophobicity. The reason for this is the large size of the macroscopic grooves, which is beyond the scale of the hydrophobic surface. Further low surface energy modification of both surfaces, the CA in both parallel and perpendicular directions of the unprocessed titanium alloy plate are increased to 96°, showing isotropic hydrophobicity, as shown in Figure 6c. After low surface energy modification of the titanium alloy surface with a macro groove structure, it exhibited a directional hydrophobic state with a measured contact angle of 135° in the parallel direction and 127° in the perpendicular direction, as shown in Figure 6d. Comparing Figure 6c,d, the surface with the macro groove structure has directional hydrophobicity after low surface energy modification, further indicating that the presence of the macrostructure is the reason for the directional hydrophobicity of the surface.

#### 3.3.2. Effect of Microstructure on Surface Hydrophobicity

**Figure 7:**
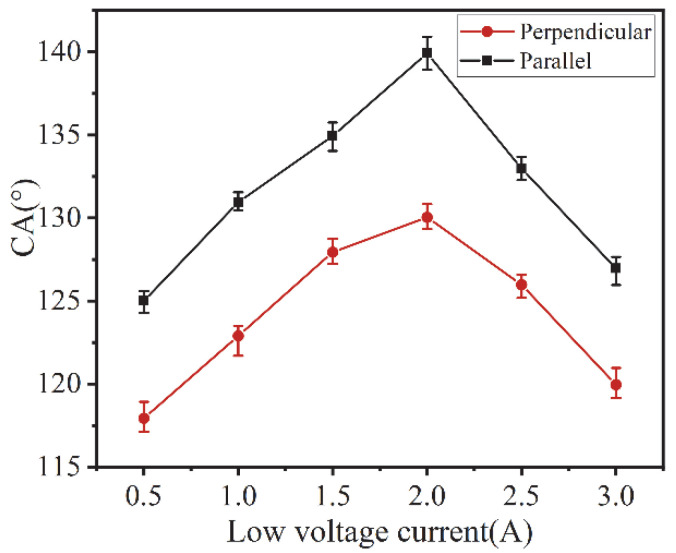
Contact of the CA of the surface of the macro-micro composite structure processed by different low-voltage electric currents.

The surface of the groove structure modified by low surface energy exhibits directional hydrophobicity, which indicates that the macroscopic groove structure is an important condition for directional hydrophobicity. The polished titanium alloy plate with grooves in Figure 6b does not exhibit hydrophobic properties, which indicates that the macroscopic groove structure itself cannot achieve the transition from hydrophilic to hydrophobic titanium alloy surfaces. In this section, the fabrication of microstructures on the smooth groove edge of macrostructures was realized by using the HV-μEAM technique, and the surface hydrophobicity of macro-micro-complex structures constructed under different low voltage currents was investigated. The directional wetting angle of the surface of the macro-micro composite structure was measured at low voltage currents from 0.5 A to 3.0 A, as shown in Figure 7.

The experimental results show that the parallel contact angle and perpendicular contact angle of the surface of the macro-micro composite structure formed under different low-voltage electric currents are greater than 90°, which exhibits hydrophobicity. Due to the macro groove structure, the parallel contact angle is larger than the perpendicular contact angle. As the low-voltage electric current increases from 0.5 A to 2.0 A, the directional contact angle increases to a maximum value, reaching 140° for the parallel contact angle and 130° for the perpendicular contact angle. When the current continues to increase, due to the excessive energy, the micro-scale structure of the groove surface showed larger craters and cracks, the surface consistency became poor, and the directional CA all showed a decreasing trend.

#### 3.3.3. Surface with Macro-Micro Composite Structure by Low Surface Energy Modification

**Figure 8:**
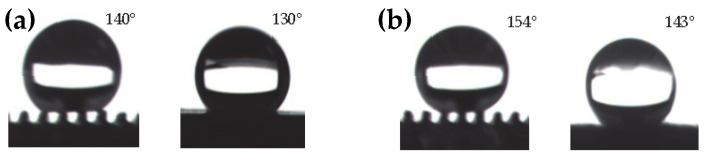
Effect of low surface energy modification on surface hydrophobicity. (**a**) CA of unmodified titanium alloy with macro-micro composite structure. (**b**) Contact angle of modified titanium alloy with macro-micro-composite structure.

Macro-micro composite structure surface formed by using the HV-μEAM technique on the macro groove edge enables the transition from hydrophilic to hydrophobic for titanium alloy plates. Figure 8a shows that the contact angle reached 140° parallelly and 130° perpendicularly after machining the microstructure on the macrostructure at a low voltage current of 2.0 A. It is due to the presence of the macro-micro composite structure that a titanium alloy directional hydrophobic surface was prepared, and its directional hydrophobic effect is superior to that of the grooves after fluorosilane modification. After the low surface energy modification on the obtained macro-composite surface, the directional contact angle was further improved, which reached 154° in the parallel direction and 143° in the perpendicular direction, as shown in Figure 8b.

**Figure 9:**
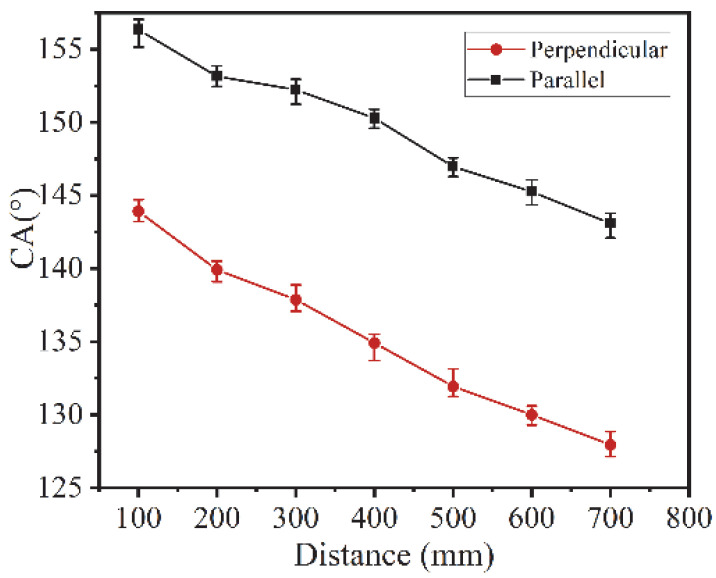
CA with different friction distances.

To explore the wear resistance of the directional hydrophobic surface of the titanium alloy, friction and wear experiments were carried out. The 600# SiC sandpaper was used, and the specimen was pushed along the direction parallel to the groove with a uniform motion at 100 mm intervals under a load of 100 g, reciprocating. The experiments were conducted in six groups, and the average values were taken. The results obtained for each contact angle are shown in Figure 9. It can be seen from the experimental results that the CA in all directions decreases with the increase of friction distance. At the friction distance of 700 mm, the parallel contact angle and perpendicular contact angle still retained the directional hydrophobicity. With the increase of friction distance, the difference between the parallel contact angle and perpendicular contact angle gradually became larger, from the initial 11° to 15°. The reason is that the macrostructure is more visible. The specimen was mechanically damaged during sandpaper friction, resulting in a decrease in contact angle. As the friction distance increases, the contact angle shows a decreasing trend. When the friction distance reaches 400, the contact angle in the perpendicular direction is 136°, and the contact angle in the parallel direction is 150°. However, the microstructures were not completely destroyed during the friction process, so the specimens did not immediately change to hydrophilic surfaces and still exhibited directional hydrophobicity.

## 4. Conclusions

We construct macro-micro composite structures on the surface of a titanium alloy by electrical discharge to realize the directional hydrophobic surface of the titanium alloy. Analogous to the microstructure of rice leaves, the sub-millimeter structures with an edge width of 150 μm, groove width of 250 μm, and depth of 250 μm were fabricated on the titanium alloy by WEDM technology, which realized the fabrication of directional hydrophobic surface macrostructure. HV-μEAM was used to fabricate micro-scale feature-size micro-structures on the processed macro-structure edges. The effect of macro-micro complex structures on directional hydrophobic surfaces was investigated, and the following conclusions were obtained.

1. The submillimeter grooves with the groove edge width of 150 μm, groove width of 250 μm, and groove depth of 250 μm were fabricated on the surface of titanium alloy by WEDM technology. The macroscopic structure is the reason for the directional hydrophobic surface of the titanium alloy.
2. HV-μEAM technology is used to prepare microstructures on the groove edge of the macrostructure. The influence of micro-scale structure on directional contact angle is obtained by analyzing the surface morphology characteristics under different low-voltage electric current conditions. A maximum parallel contact angle of 140° and a perpendicular contact angle of 130° was achieved on the surface of the titanium macro-micro composite structure.
3. After rubbing the directional hydrophobic titanium alloy specimens on sandpaper for 700 mm under a load of 100 g, the parallel contact angle and perpendicular contact angle still retained the directional hydrophobicity.

## Figures and Tables

**Table 1:** Chemical composition of TC4 titanium alloy.

Chemical Composition (wt%)							
Al	V	Fe	O	C	N	H	Ti
6.5	4.3	0.06	0.08	0.10	0.01	0.01	Bal.

**Table 2:** Four-factor and four-level orthogonal test data table.

Number	Pulse Width/μs	Pulse Interval/μs	Peak Current/A	Gap Voltage/V	Vacant Column	Edge Width /μm
1	16	30	3	5	1	112.91
2	16	35	4	6	2	114.54
3	16	40	5	7	3	93.25
4	16	45	6	8	4	106.95
5	24	30	4	7	4	145.36
6	24	35	3	8	3	146.21
7	24	40	6	5	2	100.18
8	24	45	5	6	1	92.33
9	32	30	5	8	2	89.63
10	32	35	6	7	1	91.25
11	32	40	3	6	4	93.96
12	32	45	4	5	3	96.12
13	40	30	6	6	3	90.44
14	40	35	5	5	4	77.44
15	40	40	4	8	1	115.35
16	40	45	3	7	2	87.46

**Table 3:** Four factors and four levels of orthogonal test analysis table.

	Pulse Width/μs	Pulse Interval/μs	Peak Current/A	Gap Voltage/V	Vacant Column
K_ji_	427.65	438.33	440.54	386.65	411.84
	484.08	429.43	471.36	391.26	391.80
	370.95	402.74	352.65	417.32	426.02
	370.68	382.86	388.82	458.14	423.71
$\bar{K_{\mathrm{ji}}}$	106.91	109.58	110.13	96.66	102.96
	121.02	107.36	117.84	97.81	97.95
	92.74	100.68	88.16	104.33	106.51
	92.67	95.72	97.20	114.53	105.93
R_j_	28.35	13.86	29.68	17.87	8.56

**Table 4:** Machining parameters of high voltage-induced weak arc discharge.

Parameter	Value
High voltage (V)	2000
High voltage current (mA)	0.3
Low voltage (V)	30
Low voltage current (A)	0.5/1.0/1.5/2.0/2.5/3.0
Discharge medium	Ar
Workpiece electrode (negative electrode)	TC4
Tool electrode (positive electrode)	Copper (ϕd: 500 μm)
Discharge gap (μm)	200
Scanning speed (μm/s)	15
